# Comparison of Hospitalization Rates and Clinical Features Between Boys and Girls With Respiratory Syncytial Virus Infection

**DOI:** 10.1111/irv.70235

**Published:** 2026-01-31

**Authors:** Erika Uusitupa, Matti Waris, Tytti Vuorinen, Terho Heikkinen

**Affiliations:** ^1^ Department of Pediatrics University of Turku and Turku University Hospital Turku Finland; ^2^ Department of Clinical Microbiology Turku University Hospital Turku Finland; ^3^ Institute of Biomedicine University of Turku Turku Finland

**Keywords:** children, hospitalization, respiratory syncytial virus, sex difference, wheezing

## Abstract

**Background:**

Male sex is a well‐known risk factor for respiratory syncytial virus (RSV) hospitalization in children, but there are no data on potential differences in clinical features between boys and girls hospitalized with RSV infection.

**Methods:**

We compared the average population‐based rates of hospitalization and the clinical features of the illness between boys and girls hospitalized with virologically confirmed RSV infection during 2006–2020 at Turku University Hospital, Finland. During this period, testing for RSV was routine in all children admitted with respiratory infections. The comparisons were performed in different age groups of children up to 5 years of age.

**Results:**

Among all 1204 children < 5 years of age hospitalized with RSV, the average annual RSV hospitalization rates were 4.0/1000 in boys and 3.3/1000 in girls (incidence rate ratio [IRR] 1.21; 95% CI, 1.07–1.35; *p* = 0.001). The difference was greatest in children aged 3–23 months, among whom the corresponding rates were 5.4/1000 in boys and 3.6/1000 in girls (IRR, 1.50; 95% CI, 1.25–1.80; *p* < 0.001). The occurrence of respiratory distress was consistently higher in boys than in girls among children aged 6–17 months. In this group of 233 children, 128 of 141 (90.8%) boys had documented respiratory distress, compared with 70 of 92 (76.1%) girls (*p* = 0.002).

**Conclusions:**

Except for the first 3 months after birth, boys have a 50% higher risk of RSV hospitalization than girls during the first 2 years of life. In that same age group, boys hospitalized with RSV have also significantly more respiratory distress than girls.

## 1 Introduction

Respiratory syncytial virus (RSV) is the leading cause of hospitalization for lower respiratory tract infection among infants and young children [1, 2, 3, 4, 5, 6, 7, 8, 9]. The risk of RSV hospitalization is highest during the first 6 months of life, peaking during the second month after birth [8, 9, 10, 11, 12]. Increasing knowledge about the great burden of RSV on young children has underscored the need to find preventive measures especially for the severe forms of RSV illness. Recent advances in the quest for effective interventions against RSV include an extended half‐life monoclonal antibody nirsevimab and a prefusion F vaccine administered during pregnancy [13]. Both of these approaches have shown high efficacy for reducing the burden of RSV in infants, and additional products are likely to become available in the near future [14, 15, 16, 17].

Several studies with different designs have demonstrated that male sex is a risk factor for RSV hospitalization in children [11, 12, 18, 19, 20, 21, 22, 23, 24, 25, 26]. However, most previous studies have assessed the differences in RSV hospitalization between boys and girls only in the entire age group of children < 5 years or have relied on ICD codes or clinical diagnoses without a virologic confirmation of an RSV illness. Furthermore, there are scarce data on the signs and symptoms of children hospitalized with RSV [27], and no published data on potential differences in clinical features between boys and girls hospitalized with RSV infection.

The purpose of this 14‐year study was to compare the average population‐based incidence rates and the clinical presentation between different age groups of boys and girls hospitalized with RSV infection.

## 2 Methods

### 2.1 Study Design and Population

This retrospective study was conducted at the Department of Pediatrics, Turku University Hospital, Finland, during the 14‐year period of July 1, 2006, through June 30, 2020. The study population consisted of all children < 5 years of age who were hospitalized with virologically confirmed RSV infection at the Department of Pediatrics. Turku University Hospital is the only pediatric tertiary‐care hospital in Southwestern Finland, and it is the sole provider of acute hospital care for children. To allow for conservative estimation of population‐based rates of hospitalization, only children who lived within the catchment area of the hospital were included in the analyses. The study was approved by the Institutional Review Board at Turku University Hospital.

### 2.2 Sources of Data

Children hospitalized with virologically confirmed RSV infection were identified by searching the databases of Turku University Hospital and the Department of Virology at the University of Turku. Data on patient characteristics, clinical features, management, and outcomes of all children with RSV were collected by a structured review of their medical records. Annual data on children in different age groups who lived within the catchment area of the hospital were derived from the official databases of Statistics Finland. During the 14‐year period of the study, the average number of children < 5 years of age living in the catchment area was 23,725.

### 2.3 Viral Diagnosis

Viral sampling for determination of the etiology of the illness was routine for all children hospitalized with respiratory symptoms during the study period. The virologic diagnosis of RSV was based on RT‐PCR and/or antigen detection, and identification of RSV by either method was considered to indicate an RSV‐associated hospitalization.

### 2.4 Definitions

Respiratory distress included any signs of breathing difficulty (e.g., expiratory wheezing, intercostal or jugular retraction, or tachypnea) observed during the physical examination and documented in the medical records at any time during the hospitalization. Children were considered to have an underlying medical condition if they had a chronic lung disease, congenital heart disease, immunosuppressive condition, or neuromuscular, genetic, or intellectual disorder that hampered their ability to clear mucus secretions. The duration of hospitalization was determined by the number of nights spent in the ward. In case a child was admitted and discharged during the same calendar day, the duration was recorded as 1 day. Rehospitalization was defined as an unplanned readmission due to the same illness without the disappearance of symptoms in the meantime; in these cases, the length of stay was calculated by adding up the durations of the separate stays in the hospital.

### 2.5 Statistical Analysis

The incidence rates of RSV hospitalizations in different age groups were calculated by dividing the numbers of RSV‐associated hospitalizations by the corresponding age‐specific populations and expressed as average annual rates per 1000 children. Calculation of 95% confidence intervals (CIs) for incidence rates and their ratios and testing of the differences in incidence rates between subgroups were based on the Poisson distribution. Proportions between groups were compared using the χ^2^ or Fisher's exact test, and the Mann–Whitney *U* test was used for comparing medians. Two‐sided *p* values of < 0.05 were considered to indicate statistical significance. All statistical analyses were performed with StatsDirect, version 4.0.4 (StatsDirect, UK).

## 3 Results

### 3.1 Study Population

A total of 1204 children < 5 years of age were hospitalized with virologically confirmed RSV infection during the study period. Of these, 671 (55.7%) were boys and 533 (44.3%) were girls. Among all children hospitalized with RSV infection, 555 (46.1%) were < 3 months of age and 762 (63.3%) were < 6 months of age (Table 1). A total of 1086 (90.2%) children were born full‐term, and 1115 (92.6%) did not have any underlying medical conditions; there were no differences between boys and girls.

**TABLE 1:** Characteristics of 1204 children hospitalized with respiratory syncytial virus infection.

Characteristic	No. of children (%)	
	Boys (*n* = 671)	Girls (*n* = 533)
Age (months)		
0–2	290 (43.2)	265 (49.7)
3–5	127 (18.9)	80 (15.0)
6–11	87 (13.0)	57 (10.7)
12–23	98 (14.6)	62 (11.6)
24–59	69 (10.3)	69 (12.9)
Gestational age (week)		
≥ 37	604 (90.0)	482 (90.4)
34– < 37	34 (5.1)	27 (5.1)
32– < 34	12 (1.8)	7 (1.3)
28– < 32	10 (1.5)	9 (1.7)
< 28	11 (1.6)	8 (1.5)
Underlying condition		
None	620 (92.4)	495 (92.9)
≥ 1	51 (7.6)	38 (7.1)

### 3.2 Incidence Rates of RSV Hospitalization

**FIGURE 1:**
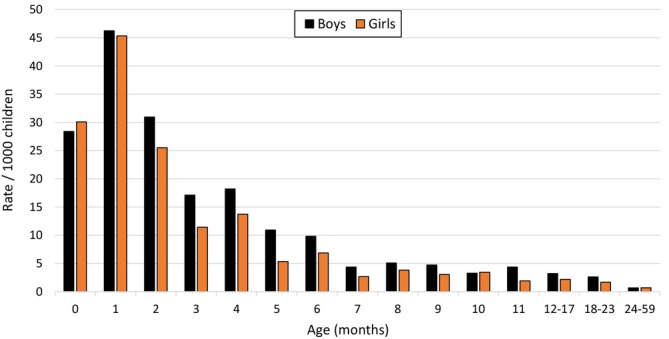
Average annual population‐based rates of RSV hospitalizations per 1000 children among boys and girls in different age groups hospitalized at Turku University Hospital, Finland, between 2006 and 2020.

The average annual population‐based rates of RSV hospitalization among boys and girls in different age groups are presented in Figure 1. Among all children < 5 years of age, the hospitalization rate was 4.0 per 1000 in boys and 3.3 per 1000 in girls, with an incidence rate ratio (IRR) of 1.21 (95% CI, 1.07–1.35, *p* = 0.001). The greatest difference between boys and girls was observed in children from 3 to 23 months of age (Table 2). In the combined age group of 3–23 months, the RSV hospitalization rates were 5.4 per 1000 among boys and 3.6 per 1000 among girls (IRR, 1.50; 95% CI, 1.25–1.80; *p* < 0.001).

**TABLE 2:** Population‐based rates and incidence rate ratios of respiratory syncytial virus‐associated hospitalizations in different age groups of boys and girls.

Age group (months)	Boys (*n* = 671)		Girls (*n* = 533)		IRR, boys/girls (95% CI)	*p*
	No. hospitalized	Rate per 1000	No. hospitalized	Rate per 1000		
0–2	290	35.2	265	33.7	1.05 (0.88–1.24)	0.60
3–5	127	15.4	80	10.2	1.52 (1.14–2.03)	0.003
6–11	87	5.3	57	3.6	1.46 (1.03–2.07)	0.03
12–23	98	2.9	62	1.9	1.52 (1.09–2.12)	0.01
24–59	69	0.7	69	0.7	0.96 (0.68–1.36)	0.80

Abbreviation: IRR, incidence rate ratio.

### 3.3 Clinical Features and Management of RSV Infection

**FIGURE 2:**
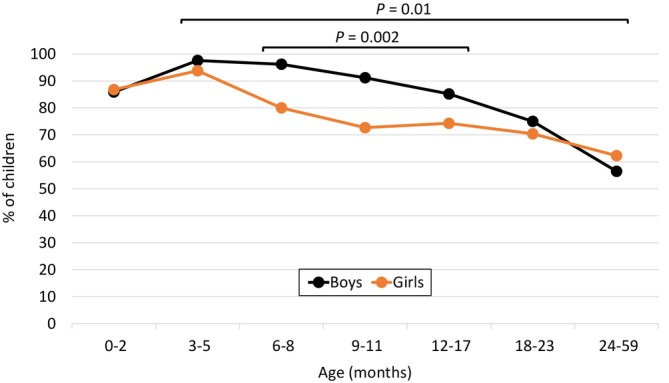
Proportions of boys and girls with respiratory distress during their RSV hospitalization. The *p* values indicate differences between boys and girls in combined age groups (6–17 months and 3–59 months).

The clinical features and management of RSV infections in different age groups of boys and girls are presented in Table 3. In the whole group of children < 5 years of age, no significant differences were observed between boys and girls in any of the measured outcomes. However, the occurrence of respiratory distress in the age group of 6–11 months was significantly higher in boys than in girls (94.3% vs. 77.2%; *p* = 0.003). To further explore this difference in more granular age groups, we halved the age groups of 6–11 and 12–23 months into 6–8, 9–11, 12–17, and 18–23 months. As shown in Figure 2, higher occurrence of respiratory distress in boys was most pronounced among children between 6 and 17 months of age. In the combined age group of 6–17 months (*n* = 233), 128 of 141 (90.8%) boys had documented respiratory distress, compared with 70 of 92 (76.1%) girls (*p* = 0.002). To rule out the possibility that the differences could be explained by the small number of children with underlying conditions, we assessed the occurrence of respiratory distress among all 1115 children who did not have any such conditions. In this analysis among previously healthy children, the differences in respiratory distress between boys and girls in different age groups followed exactly the same pattern as in the primary analysis including all children. Specifically, among previously healthy children 6–17 months of age, 116 of 127 (91.3%) boys had respiratory distress, compared with 62 of 83 (74.7%) girls (*p* = 0.001). Among all 649 children ≥ 3 months of age, 324 of 381 (85.0%) boys and 207 of 268 (77.2%) girls had respiratory distress (*p* = 0.01).

**TABLE 3:** Clinical features and management in different age groups of boys and girls hospitalized with respiratory syncytial virus infection.

Variable	Age group											
	0–2 months		3–5 months		6–11 months		12–23 months		24–59 months		0–59 months	
	Boys (*n* = 290)	Girls (*n* = 265)	Boys (*n* = 127)	Girls (*n* = 80)	Boys (*n* = 87)	Girls (*n* = 57)	Boys (*n* = 98)	Girls (*n* = 62)	Boys (*n* = 69)	Girls (*n* = 69)	Boys (*n* = 671)	Girls (*n* = 533)
Fever ≥ 39°C	9 (3.1)	7 (2.6)	17 (13.4)	12 (15.0)	28 (32.2)	21 (36.8)	48 (49.0)	35 (56.5)	42 (60.9)	34 (49.3)	144 (21.5)	109 (20.5)
Respiratory distress	249 (85.9)	230 (86.8)	124 (97.6)	75 (93.8)	82 (94.3)	44 (77.2)	79 (80.6)	45 (72.6)	39 (56.5)	43 (62.3)	573 (85.4)	437 (82.0)
Acute otitis media	125 (43.1)	111 (41.9)	65 (51.2)	38 (47.5)	48 (55.2)	37 (64.9)	47 (48.0)	24 (38.7)	26 (37.7)	17 (24.6)	311 (46.3)	227 (42.6)
Pneumonia	22 (7.6)	25 (9.4)	7 (5.5)	3 (3.8)	11 (12.6)	13 (22.8)	36 (36.7)	21 (33.9)	30 (43.5)	31 (44.9)	106 (15.8)	93 (17.4)
Antibiotic treatment	157 (54.1)	138 (52.1)	71 (55.9)	43 (53.8)	55 (63.2)	42 (73.7)	67 (68.4)	42 (67.7)	48 (69.6)	46 (66.7)	398 (59.3)	311 (58.3)
PICU admission	46 (15.9)	42 (15.8)	6 (4.7)	5 (6.3)	4 (4.6)	5 (8.8)	13 (13.3)	9 (14.5)	6 (8.7)	4 (5.8)	75 (11.2)	65 (12.2)
Rehospitalization	23 (7.9)	12 (4.5)	6 (4.7)	3 (3.8)	3 (3.4)	2 (3.5)	5 (5.1)	0 (0.0)	4 (5.8)	7 (10.1)	41 (6.1)	24 (4.5)

*Note:* Data are *n* (column %).

Abbreviation: PICU, pediatric intensive care unit.

### 3.4 Incidence Rates of RSV Hospitalization With Respiratory Distress

The population‐based rates of RSV hospitalization with respiratory distress among all children < 5 years of age were 3.4 per 1000 in boys and 2.7 per 1000 in girls (IRR, 1.26; *p* < 0.001). The IRRs between boys and girls were highest in children aged between 3 and 23 months (Table 4). In the combined age group of 3–23 months, the RSV hospitalization rates with respiratory distress were 4.9 per 1000 among boys and 2.9 per 1000 among girls (IRR, 1.66; 95% CI, 1.37–2.03; *p* < 0.001).

**TABLE 4:** Population‐based rates and incidence rate ratios of respiratory syncytial virus‐associated hospitalizations with respiratory distress in different age groups of boys and girls.

Age group (months)	Boys (*n* = 671)		Girls (*n* = 533)		IRR, boys/girls (95% CI)	*p*
	No. hospitalized	Rate per 1000	No. hospitalized	Rate per 1000		
0–2	249	30.2	230	29.2	1.03 (0.86–1.24)	0.71
3–5	124	15.0	75	9.5	1.58 (1.18–2.13)	0.002
6–11	82	5.0	44	2.8	1.78 (1.22–2.63)	0.002
12–23	79	2.4	45	1.4	1.68 (1.15–2.48)	0.005
24–59	39	0.4	43	0.4	0.87 (0.55–1.37)	0.52

Abbreviation: IRR, incidence rate ratio.

### 3.5 Rehospitalizations

A total of 65 children were rehospitalized with the same condition; 58 (89.2%) of them were readmitted within 3 days and all within 6 days of their initial discharge. Despite a trend towards a higher proportion of boys being rehospitalized in the youngest age groups (Table 3), the differences were not statistically significant.

### 3.6 Duration of Hospitalization

The median duration of hospitalization was 3.0 days (interquartile range [IQR], 1.0–4.0 days) among infants aged 0–2 months and 2.0 days (IQR, 1.0–3.0 days) in all other age groups. No significant differences were observed between boys and girls in any age groups.

## 4 Discussion

Our population‐based 14‐year analysis provides completely new quantitative data on the increased risk of RSV hospitalization in boys compared with girls. During the first months of life, the rates of hospitalization were similar in boys and girls, but from 3 months up to 2 years of age, boys had a 50% higher risk of hospitalization with RSV infection. The assessment of the clinical features of the illnesses revealed that boys had significantly more respiratory distress than girls at the age when the differences in hospitalization rates were greatest. As a consequence, the risk of hospitalization with RSV‐associated respiratory distress was 66% higher in boys than in girls among children aged 3–23 months.

When comparing the risk of RSV hospitalization between boys and girls, population‐based analyses provide the most accurate information because the birth rate of boys is approximately 5% higher than that of girls, and young infants comprise the majority of RSV hospitalizations. In previous virologically confirmed population‐based studies of unselected children < 5 years of age, the rates of RSV hospitalization were 0.7–1.0 per 1000 higher among boys than in girls [11, 12, 23], which is in line with our finding of a difference of 0.7 per 1000 children. However, the previous studies did not stratify the children by narrower age groups.

To our knowledge, this is the first study to compare the clinical features of RSV illness between hospitalized boys and girls. A recent literature review of signs and symptoms of RSV also included hospitalized children but did not compare boys and girls [27]. Although male sex has been identified for a long time as an independent risk factor for severe RSV infection and hospitalization [28], our study demonstrates for the first time that the increased occurrence of respiratory distress in boys appears to be limited to children < 2 years of age. Interestingly, however, no difference in respiratory distress or any other measure of illness severity was observed between boys and girls in the age group of < 3 months that accounts for 50%–60% of all RSV hospitalizations in infants [7, 9, 29].

The reasons and underlying mechanisms for increased severity of RSV in boys compared with girls are not fully understood although sex‐based immunological differences are known to contribute to susceptibility to infectious diseases [30]. Besides sex chromosomes, other proposed factors include sex hormones and their effect on immune responses and lung development, genetic polymorphisms, composition of the microbiome, anatomy of the airways, and diminished lung function [31, 32, 33, 34, 35]. A recent study indicated that the sex of an infant was even associated with the genotype of RSV that the infant was infected with [36]. Despite the advances in understanding the influence of sex on infections caused by RSV and many other viruses, it is obvious that susceptibility to severe disease is a complex interplay between various factors [30, 31]. Our present finding about the absence of difference in illness severity between boys and girls during the first months of life but a clear difference emerging at a later stage suggests that the impact and importance of different factors may also vary with age.

The main strengths of our study include the long 14‐year study period that balanced any variations between different seasons and routine viral sampling in all children hospitalized with respiratory symptoms. Moreover, detailed annual data on the catchment population of the hospital and restricting the analyses only to children who lived within the catchment area enabled the calculation of accurate rates of RSV hospitalizations in different age groups. There are also some limitations. Although the analyses of the clinical features were based on structured reviews of medical records, the typical limitations of this study design, including the heterogeneity of medical records, must be appreciated. Specifically, sufficiently detailed data on oxygen saturation levels and the need for respiratory support were not available for meaningful analyses. Although testing for RSV was a routine procedure, there may have been some children who missed the sampling or whose test results were false negative. However, there is no reason to assume that these limitations would have biased the comparisons between boys and girls. Even though this was a large study including more than 1200 hospitalized children, the numbers of children for some subgroup analyses were relatively small, which reduced the power to show statistically significant differences.

In conclusion, our population‐based study in different age groups demonstrates that during the first months of life, boys and girls have similar rates of RSV hospitalization, with no difference in the severity of illness. However, after the first months up to 2 years of age, boys have a 50% higher rate of RSV hospitalization than girls, which coincides with the age of increased occurrence of respiratory distress among boys. Further research is warranted to explore the underlying mechanisms of sex differences in RSV‐associated respiratory distress and the reasons why such differences are not observed during the very first months of life.

## Author Contributions

**Erika Uusitupa:** conceptualization, formal analysis, methodology, writing – original draft, writing – review and editing. **Matti Waris:** formal analysis, investigation, methodology, writing – review and editing. **Tytti Vuorinen:** formal analysis, investigation, methodology, writing – review and editing. **Terho Heikkinen:** conceptualization, formal analysis, methodology, project administration, supervision, writing – review and editing.

## Ethics Statement

This register‐based study was approved by the Institutional Review Board at Turku University Hospital. According to local research standards, patient consent or approval by the Ethics committee were not required for register‐based studies.

## Conflicts of Interest

T.H. has received honoraria for lectures or participation in advisory boards or data monitoring committees from Sanofi, Enanta, MSD, Pfizer, Shionogi, and Moderna. All other authors report no potential conflicts.

## Data Availability

The data that support the findings of this study are available on request from the corresponding author. The data are not publicly available due to privacy or ethical restrictions.

## References

[1] C. B. Hall , G. A. Weinberg , M. K. Iwane , et al., “The Burden of Respiratory Syncytial Virus Infection in Young Children,” New England Journal of Medicine 360 (2009): 588–598. 19196675 10.1056/NEJMoa0804877 PMC4829966

[2] Y. Li , X. Wang , D. M. Blau , et al., “Global, Regional, and National Disease Burden Estimates of Acute Lower Respiratory Infections due to Respiratory Syncytial Virus in Children Younger Than 5 Years in 2019: A Systematic Analysis,” Lancet 399 (2022): 2047–2064. 35598608 10.1016/S0140-6736(22)00478-0 PMC7613574

[3] C. S. Arriola , L. Kim , G. Langley , et al., “Estimated Burden of Community‐Onset Respiratory Syncytial Virus‐Associated Hospitalizations Among Children Aged < 2 Years in the United States, 2014–15,” Journal of the Pediatric Infectious Diseases Society 9 (2020): 587–595. 31868913 10.1093/jpids/piz087 PMC7107566

[4] J. M. Langley , V. Bianco , J. B. Domachowske , et al., “Incidence of Respiratory Syncytial Virus Lower Respiratory Tract Infections During the First 2 Years of Life: A Prospective Study Across Diverse Global Settings,” Journal of Infectious Diseases 226 (2022): 374–385. 35668702 10.1093/infdis/jiac227 PMC9417131

[5] R. T. Stein , L. J. Bont , H. Zar , et al., “Respiratory Syncytial Virus Hospitalization and Mortality: Systematic Review and Metaanalysis,” Pediatric Pulmonology 52 (2017): 556–569. 27740723 10.1002/ppul.23570 PMC5396299

[6] J. M. McLaughlin , F. Khan , H. J. Schmitt , et al., “Respiratory Syncytial Virus‐Associated Hospitalization Rates Among US Infants: A Systematic Review and Meta‐Analysis,” Journal of Infectious Diseases 225 (2022): 1100–1111. 33346360 10.1093/infdis/jiaa752 PMC8921994

[7] J. G. Wildenbeest , M. N. Billard , R. P. Zuurbier , et al., “The Burden of Respiratory Syncytial Virus in Healthy Term‐Born Infants in Europe: A Prospective Birth Cohort Study,” Lancet Respiratory Medicine 11 (2023): 341–353. 36372082 10.1016/S2213-2600(22)00414-3 PMC9764871

[8] C. B. Hall , G. A. Weinberg , A. K. Blumkin , et al., “Respiratory Syncytial Virus‐Associated Hospitalizations Among Children Less Than 24 Months of Age,” Pediatrics 132 (2013): e341–e348. 23878043 10.1542/peds.2013-0303

[9] E. Uusitupa , M. Waris , T. Vuorinen , and T. Heikkinen , “Respiratory Syncytial Virus‐Associated Hospitalizations in Children: A 10‐Year Population‐Based Analysis in Finland, 2008‐2018,” Influenza and Other Respiratory Viruses 18 (2024): e13268. 38477388 10.1111/irv.13268 PMC10934253

[10] R. M. Reeves , M. van Wijhe , S. Tong , et al., “Respiratory Syncytial Virus‐Associated Hospital Admissions in Children Younger Than 5 Years in 7 European Countries Using Routinely Collected Datasets,” Journal of Infectious Diseases 222, no. S7 (2020): S599–S605. 32815542 10.1093/infdis/jiaa360

[11] B. Rha , A. T. Curns , J. Y. Lively , et al., “Respiratory Syncytial Virus‐Associated Hospitalizations Among Young Children: 2015‐2016,” Pediatrics 146 (2020): e20193611. 32546583 10.1542/peds.2019-3611 PMC12874392

[12] A. T. Curns , B. Rha , J. Y. Lively , et al., “Respiratory Syncytial Virus‐Associated Hospitalizations Among Children < 5 Years Old: 2016‐2020,” Pediatrics 153 (2024): e2023062574. 38298053 10.1542/peds.2023-062574

[13] N. I. Mazur , J. Terstappen , R. Baral , et al., “Respiratory Syncytial Virus Prevention Within Reach: The Vaccine and Monoclonal Antibody Landscape,” Lancet Infectious Diseases 23 (2023): e2–e21. 35952703 10.1016/S1473-3099(22)00291-2 PMC9896921

[14] M. P. Griffin , Y. Yuan , T. Takas , et al., “Single‐Dose Nirsevimab for Prevention of RSV in Preterm Infants,” New England Journal of Medicine 383 (2020): 415–425. 32726528 10.1056/NEJMoa1913556

[15] L. L. Hammitt , R. Dagan , Y. Yuan , et al., “Nirsevimab for Prevention of RSV in Healthy Late‐Term and Term Infants,” New England Journal of Medicine 386 (2022): 837–846. 35235726 10.1056/NEJMoa2110275

[16] E. A. F. Simões , S. A. Madhi , W. J. Muller , et al., “Efficacy of Nirsevimab Against Respiratory Syncytial Virus Lower Respiratory Tract Infections in Preterm and Term Infants, and Pharmacokinetic Extrapolation to Infants With Congenital Heart Disease and Chronic Lung Disease: A Pooled Analysis of Randomised Controlled Trials,” Lancet Child & Adolescent Health 7 (2023): 180–189. 36634694 10.1016/S2352-4642(22)00321-2 PMC9940918

[17] B. Kampmann , S. A. Madhi , I. Munjal , et al., “Bivalent Prefusion F Vaccine in Pregnancy to Prevent RSV Illness in Infants,” New England Journal of Medicine 388 (2023): 1451–1464. 37018474 10.1056/NEJMoa2216480

[18] J. G. Liese , E. Grill , B. Fischer , et al., “Incidence and Risk Factors of Respiratory Syncytial Virus‐Related Hospitalizations in Premature Infants in Germany,” European Journal of Pediatrics 162 (2003): 230–236. 12647195 10.1007/s00431-002-1105-7

[19] A. Haerskjold , K. Kristensen , M. Kamper‐Jørgensen , A. M. Nybo Andersen , H. Ravn , and L. Graff Stensballe , “Risk Factors for Hospitalization for Respiratory Syncytial Virus Infection: A Population‐Based Cohort Study of Danish Children,” Pediatric Infectious Disease Journal 35 (2016): 61–65. 26398871 10.1097/INF.0000000000000924

[20] B. J. Law , J. M. Langley , U. Allen , et al., “The Pediatric Investigators Collaborative Network on Infections in Canada Study of Predictors of Hospitalization for Respiratory Syncytial Virus Infection for Infants Born at 33 Through 35 Completed Weeks of Gestation,” Pediatric Infectious Disease Journal 23 (2004): 806–814. 15361717 10.1097/01.inf.0000137568.71589.bd

[21] X. Carbonell , J. R. Fullarton , K. L. Gooch , and J. Figueras‐Aloy , “The Evolution of Risk Factors for Respiratory Syncytial Virus‐Related Hospitalisation in Infants Born at 32–35 Weeks' Gestational Age: Time‐Based Analysis Using Data From the FLIP‐2 Study,” Journal of Perinatal Medicine 40 (2012): 685–691. 23093079 10.1515/jpm-2011-0248

[22] M. T. Jepsen , R. Trebbien , H. D. Emborg , et al., “Incidence and Seasonality of Respiratory Syncytial Virus Hospitalisations in Young Children in Denmark, 2010 to 2015,” Eurosurveillance 23 (2018): 17‐00163. 10.2807/1560-7917.ES.2018.23.3.17-00163 PMC5792699 29386093

[23] S. A. Buchan , H. Chung , T. To , et al., “Estimating the Incidence of First RSV Hospitalization in Children Born in Ontario, Canada,” Journal of the Pediatric Infectious Diseases Society 12 (2023): 421–430. 37335754 10.1093/jpids/piad045 PMC10389057

[24] N. Homaira , K. A. Mallitt , J. L. Oei , et al., “Risk Factors Associated With RSV Hospitalisation in the First 2 Years of Life, Among Different Subgroups of Children in NSW: A Whole‐of‐Population‐Based Cohort Study,” BMJ Open 6 (2016): e011398. 10.1136/bmjopen-2016-011398 PMC4932307 27357197

[25] L. J. Stockman , A. T. Curns , L. J. Anderson , and G. Fischer‐Langley , “Respiratory Syncytial Virus‐Associated Hospitalizations Among Infants and Young Children in the United States, 1997‐2006,” Pediatric Infectious Disease Journal 31 (2012): 5–9. 21817948 10.1097/INF.0b013e31822e68e6

[26] P. Vartiainen , S. Jukarainen , S. A. Rhedin , et al., “Risk Factors for Severe Respiratory Syncytial Virus Infection During the First Year of Life: Development and Validation of a Clinical Prediction Model,” Lancet Digit Health 5 (2023): e821–e830. 37890904 10.1016/S2589-7500(23)00175-9

[27] A. Colosia , J. Costello , K. McQuarrie , K. Kato , and K. Bertzos , “Systematic Literature Review of the Signs and Symptoms of Respiratory Syncytial Virus,” Influenza and Other Respiratory Viruses 17 (2023): e13100. 36824394 10.1111/irv.13100 PMC9899685

[28] E. A. F. Simoes , “Environmental and Demographic Risk Factors for Respiratory Syncytial Virus Lower Respiratory Tract Disease,” Journal of Pediatrics 143 (2003): S118–S126. 14615710 10.1067/s0022-3476(03)00511-0

[29] M. Del Riccio , P. Spreeuwenberg , R. Osei‐Yeboah , et al., “RESCEU Investigators. Burden of Respiratory Syncytial Virus in the European Union: Estimation of RSV‐Associated Hospitalizations in Children Under 5 Years,” Journal of Infectious Diseases 228 (2023): 1528–1538. 37246724 10.1093/infdis/jiad188 PMC10681872

[30] S. L. Klein and K. L. Flanagan , “Sex Differences in Immune Responses,” Nature Reviews. Immunology 16 (2016): 626–638. 10.1038/nri.2016.90 27546235

[31] M. Muenchhoff and P. J. R. Goulder , “Sex Differences in Pediatric Infectious Diseases,” Journal of Infectious Diseases 209, no. Suppl 3 (2014): S120–S126. 24966192 10.1093/infdis/jiu232 PMC4072001

[32] A. Schuurhof , L. Bont , C. L. E. Siezen , et al., “Interleukin‐9 Polymorphism in Infants With Respiratory Syncytial Virus Infection: An Opposite Effect in Boys and Girls,” Pediatric Pulmonology 45 (2010): 608–613. 20503287 10.1002/ppul.21229

[33] T. Seaborn , M. Simard , P. R. Provost , B. Piedboeuf , and Y. Tremblay , “Sex Hormone Metabolism in Lung Development and Maturation,” Trends in Endocrinology and Metabolism 21 (2010): 729–738. 20971653 10.1016/j.tem.2010.09.001

[34] F. D. Martinez , W. J. Morgan , A. L. Wright , C. J. Holberg , and L. M. Taussig , “Diminished Lung Function as a Predisposing Factor for Wheezing Respiratory Illness in Infants,” New England Journal of Medicine 319 (1988): 1112–1117. 3173442 10.1056/NEJM198810273191702

[35] Y. Nagayama , T. Tsubaki , S. Nakayama , et al., “Gender Analysis in Acute Bronchiolitis due to Respiratory Syncytial Virus,” Pediatric Allergy and Immunology 17 (2006): 29–36. 16426252 10.1111/j.1399-3038.2005.00339.x

[36] Y. Tan , M. H. Shilts , C. Rosas‐Salazar , et al., “Influence of Sex on Respiratory Syncytial Virus Genotype Infection Frequency and Nasopharyngeal Microbiome,” Journal of Virology 97 (2023): e0147222. 36815771 10.1128/jvi.01472-22 PMC10062153

